# Living Labs in digital health: a collaborative ecosystem approach for continuum of care

**DOI:** 10.3389/fpubh.2025.1728904

**Published:** 2026-01-06

**Authors:** Tiziana Russo-Spena, Claudia Salvatore, Beth Fairfield, Annachiara Giordano, Serena De Simone, Maddalena Illario

**Affiliations:** 1 Department of Economics, Management, Institutions, University of Naples Federico II, Naples, Italy; 2 Department of Public Health, University of Naples Federico II, Naples, Italy; 3 Department of Humanities, University of Naples Federico II, Naples, Italy

**Keywords:** Living Labs, digital health, systematic review, health service provision, collaborative ecosystems

## Abstract

**Introduction:**

Living Labs (LLs) are increasingly recognized in healthcare as collaborative environments that drive digital health innovation through stakeholder co-creation in real-world settings. However, existing research has predominantly conceptualized LLs as experimental testbeds for technology validation, neglecting their broader systemic potential in integrated health services.

**Objectives:**

This study seeks to advance the understanding of LLs in digital health by identifying their foundational elements, analyzing their methodologies, and proposing an expanded framework for their application within healthcare ecosystems.

**Methods:**

The study combines a systematic scoping review, guided by PRISMA and based on ENOLL's (2021) six LL principles, with expert focus group discussions. This multi-method design integrates conceptual insights with practical perspectives from digital health contexts.

**Findings:**

The study delineates the defining features of LLs in digital health, including their typologies, methodological specificities, stakeholder configurations, intended outcomes, and systemic challenges. These insights inform the Collaborative Living Ecosystem Approach (CLEA), a conceptual framework that reconceptualizes LLs as multidimensional platforms for sustainable healthcare innovation.

**Discussion:**

CLEA encompasses three core dimensions: a reconceptualization of LLs beyond innovation hubs, a stakeholder-driven model that fosters co-creation, and an emphasis on integrating healthcare services to enhance connectivity across health and care levels. By providing a structured and integrated perspective on LLs, this study contributes to the discourse on digital health ecosystems, shifting the focus from technology validation to holistic, sustainable healthcare solutions that address complex health and societal needs.

## 1 Introduction

The Living Labs (LLs) concept, introduced by Professor William Mitchell at MIT's MediaLab, represents a groundbreaking innovation in research methodologies. A Living Lab is fundamentally defined as an open innovation ecosystem that fosters collaboration among a diverse set of stakeholders, including end-users, businesses, research institutions, and public entities, to co-create solutions that are technically robust, socially relevant, and economically viable (1). Initially applied in urban planning and smart home technologies (2), the Living Lab framework has evolved to address complex societal challenges across various sectors, including healthcare.

The health sector has increasingly adopted the Living Lab (LL) approach as a strategic framework to confront complex systemic challenges (3), including the enhancement of patient outcomes, the optimization of care delivery efficiency, and the need to respond effectively to demographic shifts such as population aging and the rising prevalence of chronic diseases (4). This approach facilitates the adaptation and integration of innovative digital solutions that promote a person-centered and anticipatory model of healthcare delivery. The European Network of Living Labs (ENoLL), comprising over 150 active LLs worldwide, exemplifies the success of the Living Lab approach in bridging the persistent gap between academic research and real-world applications, particularly in domains such as digital health. Prominent cases illustrate the flexibility and relevance of LLs in fostering health innovation. For example, the “Digital Health Living Lab” in the United Kingdom has played a pivotal role in the development of personalized health solutions. At the same time, the “Living Lab for Aging and Wellbeing” in the Netherlands addresses the specific needs of aging populations through participatory and user-centered innovation. In the Italian context, initiatives such as the City of the Future Living Lab and Trentino Salute 4.0 (TS4.0) demonstrate the capacity of LLs to align local policymaking, healthcare provision, and scientific research in the pursuit of sustainable and context-sensitive solutions.

By embedding research and development within real-world environments and leveraging iterative, co-creative processes, Living Labs offer a distinctive and dynamic platform for the design, evaluation, and implementation of digital health innovations. These innovations are tailored to reflect the needs, preferences, and lived experiences of patients, healthcare providers, and other key stakeholders (4).

Despite the demonstrated versatility and impact of Living Labs (LLs), academic discourse has predominantly emphasized their role in facilitating targeted innovations and addressing discrete health challenges. Nevertheless, several critical dimensions of their broader transformative potential remain undertheorized and insufficiently explored. LLs have traditionally been conceptualized as problem-oriented instruments. However, they are increasingly aligned with emerging public health paradigms that conceptualize health as a technologically mediated, person-centered phenomenon shaped by the dynamic interplay of physical, mental, and social environments (4).

In response to this paradigm shift, there is a pressing need to reconceptualize LLs as systemic infrastructures capable of fostering cross-sectoral integration and sustaining long-term stakeholder engagement. Crucially, LLs should be positioned to support the co-creation of transformative solutions to the complex and evolving challenges confronting contemporary health systems.

To address this conceptual and empirical gap, the present study investigates the evolving role of LLs in the context of health system transformation. The following questions guide the research: *RQ1: What is the current state of knowledge regarding LLs in health within the scientific literature? RQ2: How can insights derived from literature inform the development of future LL frameworks for integrated health services?*

By shifting the analytical focus beyond the validation of technological innovations, this study seeks to elucidate the potential of LLs as holistic frameworks capable of addressing the multidimensional nature of health. These aspects include promoting preventive care, fostering patient- and citizen-centered innovation, and enhancing societal impact.

This study employs a multi-method approach combining a systematic scoping literature review with focus group discussions to integrate theoretical insights and practical expertise (5). The systematic review followed the PRISMA (Preferred Reporting Items for Systematic Reviews and Meta-Analyses) guidelines to ensure methodological transparency and replicability, utilizing PubMed, Scopus, Web of Science, and Google Scholar. Insights from this review were further enriched by engaging experts through focus groups, which provided nuanced, real-world perspectives on LLs' operational challenges and transformative potential in health.

This study synthesizes insights into LLs application contexts, stakeholder engagement practices, and associated outcomes while identifying key challenges. Building on these insights, it advances the conceptualization of LLs in health by introducing the Collaborative Living Ecosystem Approach (CLEA), a framework that redefines LLs as integrated, multidimensional healthcare innovation platforms that combine research, health innovation, and service improvement to drive sustainable transformation in healthcare. CLEA promotes integrated service innovation grounded in real-world health needs, fosters a stakeholder-driven approach, and introduces a structured evaluation framework to assess methodological advancements, innovation-driven applications, and systemic impacts. By doing so, this framework strengthens the role of LLs in integrating digital health solutions across the continuum of care.

This paper is structured as follows: section 2 outlines the current state of the art regarding Living Labs in health. Section 3 describes the multi-method approach, combining a systematic scoping review and expert focus groups. Section 4 presents the findings, synthesizing the main themes from the literature. Section 5 introduces the Collaborative Living Ecosystem Approach (CLEA), developed through the integration of literature insights and expert reflections, and elaborates its conceptual foundations and practical implications. Finally, section 6 discusses conclusions, policy implications, and directions for future research.

## 2 Living lab and health: the state-of-the-art

The concept of Living Lab is characterized by a lack of consensus, with varying interpretations and definitions permeating the literature. Scholars propose a range of perspectives on what constitutes a Living Lab, reflecting its multifaceted nature and the diverse applications within various sectors. Some studies focus on leadership structures and primary objectives of LLs. For instance, utilizer-driven LLs are led by businesses and prioritize market-oriented innovation (6). At the same time, enabler-driven LLs, initiated by public entities or NGOs, focus on addressing societal challenges (7). Research institutions lead provider-driven LLs, emphasizing research and education (8). In addition, user-driven LLs are steered by user communities, placing user-centred co-creation at the forefront (2). Despite their differing goals, these LLs share several commonalities summarized in the six main principles of the LL approach (9). These include a focus on innovation activities through an experimental approach in real-world settings, often grounded in a multi-method approach that implies strong user engagement, multi-stakeholder participation, and the collaborative co-production of knowledge or co-creation.

LLs have emerged as critical platforms for fostering health innovation, particularly in the digital era (10, 11). They play a pivotal role in developing and evaluating digital health technologies (7), accelerating the adoption of practical solutions by iteratively testing and refining them to align with patient preferences, needs, capacities and experiences. This approach also facilitates the development of innovative healthcare models and processes, including chronic disease management, home-based care, and preventive health initiatives. Furthermore, LLs influence health policy and governance by engaging policymakers and regulators in innovation, ensuring that emerging solutions meet regulatory standards and align with broader health policy objectives (12).

A growing number of literature reviews have recently examined the functioning of Living Labs (LLs) within the health sector. Some reviews adopt a targeted focus, exploring specific contexts such as dementia care (13) or initiatives aimed at supporting older adults (14, 15). Others take a broader approach, evaluating the potential applications of LLs across diverse healthcare settings (16, 17). Additionally, some studies investigate the relationship between the LL approach and the effective implementation of healthcare innovations (11). Despite these contributions, much of the existing literature falls short of fully capturing the multifaceted nature of LLs—particularly their potential for extensive stakeholder engagement and their capacity to catalyze systemic transformation in health. For instance, while Kim et al. (10) highlight the complex roles of stakeholders in LLs, their analysis does not sufficiently examine how stakeholder involvement contributes to the overall success and sustainability of LL initiatives. Moreover, literature tends to concentrate on LLs as project-specific innovation initiatives, often focusing on their short-term outcomes rather than their effective implementation as sustainable models in health practice. This narrow scope overlooks the structural and systemic mechanisms that enable LLs to function as long-term innovation infrastructures rather than temporary experimental spaces. Consequently, a systematic examination of how LLs operate within the broader digital health ecosystem is needed. Key areas requiring attention include the impactful intervention of digital health, good practices for stakeholder engagement and the long-term sustainability of LLs. Addressing these gaps is critical for unlocking the full transformative potential of LLs in advancing health innovation and the continuum of care (cf. Table 1).

**Table 1:** A literature review on LLs in Health.

**References**	**Methodology**	**Databases & years**	**Main focus**	**Main gap**
Figueiredo et al. (13)	Narrative Literature review & case study	PubMed, Web of Science, Scopus, EBSCOhost (years are not reported)	Digital health LLs for dementia	Limited exploration of broader applications beyond specific health conditions.
Cyr et al. (16)	Scoping review	Scopus, Web of Science, MEDLINE, EMBASE, PSYCHINFO, and the EBSCO host database (2011–2021)	User engagement in HLLs	A narrow focus on user engagement strategies overlooks other critical success factors in LLs.
Kim et al. (17)	Integrative literature review	PubMed, Embase, Cinahl, PsycINFO (2000–2019)	LLs and successful health innovation implementation	Incomplete evaluation of the full spectrum of implementation outcomes in health LLs.
Verloo et al. (14)	Scoping review	Not specified (2021)	LL activities for older adults with dementia	Narrow population focus, lacking broader applicability across diverse demographic groups.
Knight-Davidson et al. (15)	Scoping review	CINAHL, Medline, PubMed, Scopus (up to 2020)	Co-creation methods with older adults	Specific focus on older adults, limiting generalisation to other population segments.
Kim et al. (10)	Integrative literature review	Broad search, health-related databases (up to 2019)	LLs as platforms for health innovation	Insufficient analysis of stakeholders' evolving roles and contributions in health innovation projects.
Zipfel et al. (11)	Integrative Literature Review	PubMed, EMBASE, PsycINFO, Cinahl (2000–2019)	Relationship between LLs approach and the successful implementation of healthcare innovations	Limited in capturing the full range of implementation outcomes based on a literature exploration.
*Our paper*	*Systematic scoping review*	*Web of Science, Scopus, Google Scholar, PubMed, Embase, CINAHL, and PsycINFO (up to December 2023)*	*LLs as platforms for integrated service for health*	–

## 3 Methods

This study employed a structured and multi-step approach to comprehensively address the research objectives. First, a systematic scoping review (18) was conducted to map the existing literature on LLs in digital health, clarify key concepts, and identify key themes. Building on these findings, a focus group with digital health experts was held to explore the themes in depth and examine their interconnections, offering deeper insights and contextualizing the literature within practical settings (19).

### 3.1 Systematic review process

**Figure 1:**
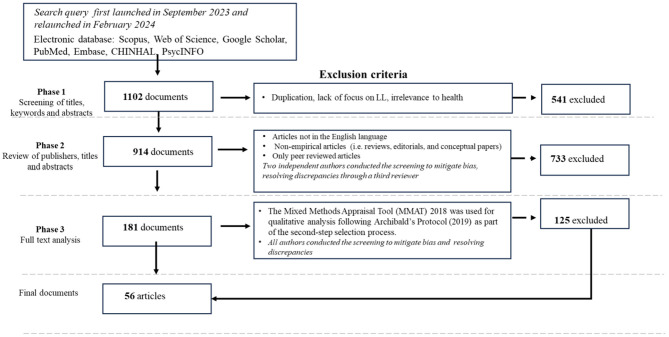
Prisma.

As the first step, the study systematically explores and synthesizes the existing literature on LLs within digital health contexts. The primary objective is to analyze their nature and roles while identifying critical knowledge gaps to guide future research and practice. To achieve this, the study is structured around three core sub-questions. RQ 1.1 *How are LLs defined and conceptualized within digital health settings?* This inquiry examines their distinctive features, particularly their integration into real-life environments and their contributions to fostering health-related innovations. The second question investigates RQ 1.2: *What unique practices characterize stakeholder involvement in LLs?* It focuses on how LLs differ from other innovation methodologies, emphasizing their capacity to engage diverse stakeholders and address broader determinants of health. The third question addresses RQ 1.3: What are the key outcomes and impacts *of LL in digital health, and what challenges and barriers affect their effectiveness*? It considers issues such as the integration of LLs' services, the sustainability of their initiatives, and the scalability of their innovations. A systematic scoping review was chosen for its ability to provide a structured and rigorous synthesis of a broad and diverse body of literature (20). Unlike traditional scoping reviews (18), this approach ensures a transparent and replicable process, essential for an emerging and interdisciplinary field like digital health, where LLs often involve predominantly qualitative or descriptive studies (20). The search strategy was developed in accordance with established systematic review guidelines to ensure both comprehensiveness and precision. Adherence to the PRISMA (Preferred Reporting Items for Systematic Reviews and Meta-Analyses) guidelines ensures a rigorous and transparent literature review (21). This thorough approach enhances the reliability of the findings and supports the validity of the study conclusions (see Figure 1).

The review began with a comprehensive search of relevant literature across multiple electronic databases, including Web of Science, Scopus, Google Scholar, PubMed, Embase, CINAHL, and PsycINFO. Given the diverse terminology used by scholars and professionals to describe the living lab phenomenon, the search included multiple keywords, such as “living lab^*^”, “living laboratory”, “living laboratories”, and “living labbing”. For health categories, the search incorporated terms like “health”, “healthcare”, and “care”. Regarding digital health, the query included keywords such as “electronic health”, “telemedicine”, “telecare”, “telehealth”, “mobile health”, “mHealth”, “e-health”, “digital medicine”, and “home care”. This query was explicitly designed to capture a wide range of studies focusing on stakeholder engagement within the context of digital health innovations (22). The initial search, conducted in September 2023 and updated in February 2024, identified 1,102 documents published up to December 2023.

Following a preliminary screening of titles, keywords, and abstracts, 541 documents were excluded due to duplication, lack of relevance to LLs, or health. This step reduced the pool to 914 studies. Selection proceeded in two phases. The first phase screened publishers, titles, and abstracts, prioritizing peer-reviewed empirical studies in English while excluding non-empirical research, such as reviews and editorials, to ensure a focus on concrete data and outcomes. Two independent authors conducted this screening, resolving discrepancies with a third reviewer, and ultimately excluded 733 articles, leaving 181 for further analysis.

In the second phase, a quality assessment of included studies was conducted using the Mixed Methods Appraisal Tool (MMAT) 2018, a validated instrument for evaluating qualitative, quantitative, and mixed-methods research. Its selection followed Archibald's (20) protocol, ensuring methodological rigor and inclusivity. The MMAT assesses three core criteria, rated as “yes”, “no”, or “cannot tell”, allowing for the inclusion of studies with well-defined research questions without imposing excessively stringent thresholds. This approach ensured a broad representation of LLs‘ activities and findings. Three authors independently assessed the documents to ensure consistency and reliability, with discrepancies resolved through collective discussion. This process led to the inclusion of 56 studies in the qualitative synthesis, offering a broad representation of LLs' activities and findings. Building on this foundation, the data extraction phase was designed to capture and organize the complexity of the selected studies in a coherent and structured manner. To address the substantial heterogeneity in study designs, contexts, and focus areas, the same three authors conducted the extraction independently, drawing on a predefined grid based on shared analytical dimensions. These included research objectives, methodological approaches, intervention settings, stakeholder roles, technological applications (e.g., IoT and sensors), outcomes, and challenges. This structured process supported both the subsequent thematic analysis and narrative synthesis, providing a robust foundation for identifying cross-cutting patterns and emerging themes.

### 3.2 Data analysis

The thematic analysis followed the structured approach outlined by Thomas and Harden (23), which involves systematic coding and synthesis of qualitative data. The coding process was iterative, allowing for refinement and adjustment as new insights emerged. For example, text segments describing stakeholder roles in LLs and activities were initially coded under specific categories, such as “stakeholder participation”, which were later grouped into the broader theme- stakeholder engagement and practices – representing the main topic of interest across the different studies, ensuring that the synthesis captured the richness of the data. Similarly, the analysis proceeded with other themes to ensure that they were directly aligned with our research sub-questions: how LLs were defined and conceptualized within health settings, the distinctive features of stakeholders' engagements, the primary outcomes and the challenges and barriers they face.

To contextualize the findings, the study adopted the narrative synthesis approach proposed by Popay et al. (24). This method went beyond identifying themes by integrating them into a cohesive narrative that detailed their interactions and implications within the broader framework of digital health innovation. For instance, stakeholders and their roles were analyzed within the narrative synthesis to explore how these elements influenced their roles. This approach highlighted the dynamic relationships between themes, offering a new framework for understanding the intricacies of LLs and their crucial role in integrating digital interventions within the evolving landscape of broader health goals. The six foundational principles of the European Network of Living Labs (ENoLL) (9) were used as an initial guide to systematically construct a conceptual structure defining the nature of Living Labs.

This preliminary framework was further explored and refined through two focus group sessions with experts in digital health, which supported the development of a context-sensitive conceptual model of Living Labs oriented toward health service integration. Each session lasted approximately 90 minutes and followed established protocols for qualitative focus group research (19), ensuring methodological consistency and depth. The groups were composed of seven participants representing a range of roles and experiences in digital health innovation: two clinical physicians (one with expertise in geriatrics, one in primary care), one biomedical engineer, one digital health researcher, one manager from a regional health authority, and two informal caregivers (one supporting an older adults parent, the other a chronically ill partner). Participants varied in age (35–68 years) and gender (four female, three male), contributing diverse perspectives across professional and personal domains. A semi-structured interview guide was used to ensure consistency across sessions while allowing for emergent insights. The guide covered topics such as stakeholder engagement strategies, integration of LLs into existing health systems, evaluation of health outcomes, and perceived barriers and enablers to scaling LL-based interventions. The discussions were audio-recorded and transcribed verbatim. The focus of the interpretation was to deepen and consolidate the thematic areas identified in the literature review, clarify how they relate to one another, and integrate participants' perspectives into a coherent synthesis. This process highlighted both convergence and divergence in viewpoints, enriching the development of the proposed framework.

## 4 Findings

This section outlines the primary aspects emerging from the literature analysis to enhance understanding of LLs in digital health. It describes the typologies and distinctive features of health-driven innovation LLs. Additionally, it explores the methodologies used, the engagement of diverse stakeholders, and the practices and activities in which they participate. Finally, it presents the key identified LLs outcomes, the resulting LLs challenges, and areas for further research.

### 4.1 Core concepts, typologies, and health-driven innovations in LLs

The findings from the systematic literature review reveal a detailed classification of the analyzed studies based on their primary focus: whether they concentrate directly on the concept of LLs or employ LLs as a methodological approach. Of the 56 articles reviewed, 27 specifically examined the idea of LLs, highlighting their effectiveness in involving end-users in the co-creation process—the remaining 29 articles employed LLs as a methodological framework for their research. The analysis also explored the distinguishing features of LLs, examining how they are defined and conceptualized in health, their fields of application within digital health, the innovations implemented and geographical distribution.

Regarding definitions (see Supplementary Table 1), LLs are most characterized as open innovation ecosystems (25, 26), a perspective that emphasizes their ability to foster collaborative innovation and experimentation among multiple stakeholders, including researchers, industry actors, and end-users. A common interpretation also frames LLs as *innovation networks*, highlighting these ecosystems as interconnected and dynamic processes (27, 28). A significant body of literature refers to the European Network of LLs (ENoLL) definition, highlighting its *user-centred approach*, which emphasizes the integration of user needs and feedback throughout the innovation lifecycle (29–31). Additionally, some studies mention the *LLs environment* as a shared collaboration space designed to replicate users' real-life contexts, including hospitals, health facilities, and private home settings (32). Some of these works also explore this concept within virtual or digital environments, further expanding its applicability and reach (28, 33).

LLs are increasingly prevalent in *primary care*, as evidenced by numerous studies (33, 34) (see Supplementary Table 2). These LLs often focus on addressing various *chronic diseases*, a significant area of concern in health. Research highlights their application in managing diabetes, cardiovascular diseases, and other long-term health challenges (35–37). In addition, studies focus on the role of LLs in *preventive health*, including fall risk prevention and alcohol misuse prevention (38, 39), as well as promoting psychological well-being and addressing mental health disorders, such as depression and anxiety, alongside broader psychological and social interventions (40, 41). Furthermore, *emergency medical situations*, including acute care scenarios, have also been explored within the LLs framework, emphasizing their adaptability to critical health challenges (27, 42). Beyond these specific applications, some studies explore LLs without anchoring them to a particular medical context. These works focus on integrating smart technologies, overarching principles, and guidelines for implementing LLs in diverse health settings. They highlight the potential of LLs as a testbed for innovative solutions, emphasizing technological advancements and collaborative approaches (43, 44).

The health-related innovations emerging from LLs primarily focus on developing new technological solutions (see Supplementary Table 3). These innovations include *mobile applications* (25, 45) and *exergames and gamified exercises* (31, 46), *software platforms* (47, 48), *wearable devices and sensors* (49–51), as well as *robotics and chatbots* (28, 52, 53). Additionally, several studies propose using LLs to develop novel *methodologies, protocols, and procedures*. These methodologies encompass diverse applications, such as enhancing medication adherence through tailored interventions (26), facilitating recruitment, surveys, and early-stage testing via collaborations between citizens and institutions or companies (54), and implementing integrated health models, such as the Epital Care Model (ECM) for remote monitoring and self-management of chronic diseases (55).

Most of the analyzed publications focus on the operations of LLs based in Europe (56, 57). These studies document LLs experiences in countries such as the Netherlands (26, 58), Germany (30, 46, 59), France (49, 60), and other Central European nations. Additionally, a smaller subset of articles explores LLs initiatives in Asia, specifically in South Korea (53, 61) and the Americas, primarily in the United States and Canada (42, 51).

### 4.2 Framework and methods

The literature analysis reveals a diverse array of theoretical frameworks and methodologies underpinning the design and implementation of LLs. Among frameworks are *User-Centered Design* (UCD) and *participatory approaches*, which emphasize co-creation and collaborative design processes (31, 45, 62). *Open Innovation* and *Technology Adoption*, focusing on frameworks such as UTAUT and Technology Acceptance Models, are also identified to explore factors influencing user acceptance and the adoption of LLs' outputs (46, 63). A prominent theme emerging from the analysis is the integration of the *Quadruple* and *Quintuple Helix frameworks*, which expands the discourse to encompass ecological sustainability (44, 48, 57) and aligns with the principles of the circular economy (58). In some studies, this integration further emphasizes sustainable and ethical engagement alongside the promotion of health equity through various initiatives (4, 14, 43), including those led by the World Health Organization. *Sociocultural* and *psychological frameworks* also contribute to the depth of user experience design in LLs, drawing on perspectives from social-cognitive theory, cultural psychology, and wellness paradigms (33, 53, 64). Finally, advanced technological frameworks play a pivotal role in the multidisciplinary approach of LLs. These frameworks incorporate *systems thinking* (65) alongside cutting-edge technologies, such as the Internet of Things (IoT), edge computing, and behavioral sensing, to improve health outcomes and quality of life (47, 66, 67).

Qualitative methodologies, including *grounded theory, thematic and phenomenological analysis, participatory research, and ethnographic methods*, dominate studies that explore user experiences and ethical considerations, reflecting the centrality of human-centred inquiry in LLs contexts (37, 41, 58). Quantitative approaches, including *surveys, physiological assessments, and comparative studies*, provided structured data to measure usability, health outcomes, and the effectiveness of interventions (30, 38, 61, 67). In addition, experimental designs, such as *randomized controlled trials and pilot studies*, underscore the commitment to rigorously testing interventions in real-world settings (33, 42, 55, 59). *Mixed methods* integrate user feedback with quantitative assessments, enhancing the robustness of iterative co-creation processes (4, 45, 52).

### 4.3 Stakeholders' roles and collaborative practices

The analysis of stakeholder roles and collaborative activities in LLs underscores the complexity and inclusivity of these ecosystems, highlighting the diverse contributions of various actors.

Patients are central to these ecosystems, whose lived experiences and contextual knowledge form the foundation for user-centred and inclusive solutions (see Supplementary Table 4). Beyond patients, other key stakeholders are identified as primary users, including *general users* (46, 68), *caregivers* (41, 69), and *vulnerable group*s (29, 44). Among professionals, the roles of *healthcare practitioners* (34, 59, 70), *designers and developers* (38, 58), and *researchers* (4, 71) are particularly emphasized. Other relevant categories include healthcare organizations, such as *healthcare facilities* (43, 49), *educational institutions* (32, 72), and *research centers* (57, 73). Some studies explicitly reference *policymakers* (44, 55), *governments and regulatory authorities* (54, 73), *communities* (42, 66, 74), and *citizens* (34).

These stakeholders participate in various co-creation activities, which can be identified in four main stages: *co-design, co-development, co-testing, and co-evaluation*. During these stages, collaboration becomes critical to the success of LLs initiatives (see Supplementary Table 5).

*Co-design practices* employ inclusive methodologies that enable stakeholders to ideate and frame innovative solutions collaboratively. Participatory workshops emerge as a central tool for fostering joint ideation, providing a structured yet open environment for brainstorming and insights generation. For instance, workshops have been instrumental in creating strategies for promoting healthy behaviors (65) and tailoring mobile applications for patients (46). Contributions from marginalized groups facilitated through discussions or stakeholder mapping (40) enrich these processes, ensuring that solutions address diverse societal challenges (74). In parallel, healthcare professionals, designers and usability experts collaborate through workshops to ensure accessibility, leveraging iterative feedback to refine ideas into actionable insights (26). Policymakers also contributed to these activities by addressing structural challenges (44) or designing frameworks and policies to ensure long-term impacts (73).

*Co-development practices* prioritize participatory approaches that bring stakeholders together to prototype and refine solutions. Iterative design cycles, including scenario-based methodologies and participatory service design, are widely applied to integrate user preferences and technical requirements (45, 48). For instance, researchers and clinicians have collaboratively developed emergency response systems for smart homes, ensuring that the technologies meet functional and clinical standards (42). Similarly, the co-development of companion robots involved interdisciplinary teams, including technical experts, who integrated cognitive and ergonomic considerations to enhance usability (53). Healthcare providers further supported these processes by aligning innovations with resource constraints and scalability objectives (68).

*Co-testing practices* focus on validating solutions through real-world experimentation and simulated scenarios, ensuring their functionality and usability. End users, particularly patients, actively participate in trials of wearable devices, providing critical feedback on reliability and usability while biomedical engineers assess technical accuracy (51). Behavioral observations, often facilitated by sociologists and anthropologists, complement these evaluations by providing contextual insights into user interactions (41). Simulations, such as VR interventions, exemplify how stakeholder collaboration can refine developmental tools for targeted populations (39). Small and medium-sized enterprises (SMEs) and technology providers play a pivotal role in bridging innovation with market readiness, ensuring solutions are tailored to diverse operational contexts (63).

Evaluation practices involve systematic assessments to optimize LLs solutions based on user feedback and performance metrics. The literature emphasizes the role of iterative evaluations in refining eHealth tools, apps, and assistive devices (45, 65). For instance, older adult participants evaluated the usability of assistive technologies, contributing to iterative improvements (49), while caregivers and healthcare practitioners identified barriers to implementation and proposed enhancements. Risk assessments, facilitated by partner organizations and institutional stakeholders, ensure solutions adhere to safety and usability standards, promoting inclusivity and scalability across diverse settings (64). Performance data, such as heart rate variability metrics, further validate device reliability and guide refinements to mobile health applications (51).

### 4.4 Main outcomes

While LLs are designed to foster the development of innovative solutions, reported outcomes vary considerably in scope and impact, spanning different stages of the innovation process (see Supplementary Table 6). Many studies focus on the development phases (27, 33, 41, 57), while others focus on the implementation phase (31, 44, 75). Few studies investigate the application of LLs components in user practices (26, 48).

Concerning outcomes, the versatility of Living Labs as an open innovation framework lies in their capacity to support the *advancement of health research*, facilitate service development, and drive health transformation. A key contribution of Living Labs lies in their role in advancing health research methodologies. Several studies emphasize their impact on refining co-creation techniques and iterative design, ensuring that health interventions align with user needs and practical applications (4, 41, 75). Living Labs facilitate the collection of rich, real-world data, combining qualitative insights from user interactions with quantitative health metrics (33, 72), thereby ensuring the feasibility and impact of interventions. Moreover, interdisciplinary collaboration fosters innovation by engaging researchers, practitioners, and patients in co-developing solutions tailored to complex healthcare challenges (31, 45). Beyond their research role, Living Labs play a pivotal role in driving *healthcare innovation*. These environments enhance service quality and patient outcomes by facilitating optimized health coordination (76), integrating advanced technologies, and streamlining healthcare processes (39, 51). Moreover, Living Labs actively foster patient engagement (33, 37) and empower individuals to take an active role in their healthcare, ultimately improving health outcomes and compliance (25, 55).

Additionally, they drive e-health innovation by incorporating digital health solutions and data-driven strategies, thereby advancing ageing-in-place initiatives and preventive care (35, 44). Beyond these functions, Living Labs *drives systemic health transformation*, shaping resilient and inclusive healthcare systems. They enhance access to healthcare, particularly for underserved populations (37, 65), while fostering social participation and empowering marginalized communities through participatory engagement (31, 60). Moreover, they promote health literacy and develop educational programs and training initiatives to facilitate the adoption of digital health (4, 41). Furthermore, some studies have demonstrated their role in supporting policy development and business growth, influencing healthcare policies, clinical guidelines, and service delivery models through real-world evidence (40, 43).

Across various studies, multiple implementation outcome measures have been identified, although emphasis on specific aspects varies (see Supplementary Table 8). Most studies emphasize the acceptability (30, 77) and appropriateness (31, 44) of LL methods and approaches. Additionally, several studies report positive results regarding the effectiveness and efficiency of innovative solutions (38, 51). Other studies highlight end-user satisfaction (26, 78) and appreciation for the functionality of LLs (37, 79). Furthermore, aspects such as safety (71) and equity (32, 48, 77) have also been investigated.

### 4.5 Challenges and research priorities

Despite their transformative potential, LLs face several persistent challenges (see Supplementary Table 9). Ensuring digital health solutions are *accessible and user-friendly* remains a significant challenge, particularly for older users (46, 58) and vulnerable populations (26, 45). Furthermore, *stakeholder engagement* is often complicated by conflicting interests, unclear roles, and difficulties fostering effective collaboration among multidisciplinary teams (48, 63). Securing *long-term funding* and ensuring *financial sustainability* also pose significant hurdles, as many LLs struggle to transition from pilot phases to fully operational healthcare solutions (29, 56). Additionally, concerns *regarding privacy*, informed consent, and *ethical guidelines* continue to be a pressing issue, necessitating more explicit frameworks for responsible data use and participant protection (31, 69). Similarly, the *scaling* and *integration* of living lab outcomes into broader healthcare systems remains complex, requiring robust policy support and regulatory alignment (35, 44).

In response to these challenges, the analyzed studies identify as research priorities (see Supplementary Table 10) the integration of *longitudinal studies* to assess long-term effectiveness and scalability (42, 68) and the development of *scaling methodologies* to ensure successful innovations extend beyond pilot projects into broader healthcare systems (58, 77). *Economic feasibility* and *cost-effectiveness* remain critical areas of inquiry, essential for evaluating the financial sustainability of living labs (56, 64). Advancing *AI-driven healthcare* solutions is also a priority, particularly in enhancing predictive analytics, patient monitoring, and decision-making (29, 52, 78). However, these innovations must align with inclusive digital health strategies to promote *health literacy* and *digital equity*, particularly among marginalized populations (25, 51). Finally, *policy development* must keep pace with technological advancements, ensuring that regulatory frameworks facilitate, rather than hinder, the integration of living labs into healthcare systems (32, 43).

## 5 Discussion

This study advances the conceptualization of Living Labs (LLs) beyond their traditional characterization as open innovation ecosystems by identifying their core elements and extending their relevance to digital health. The literature analysis clarifies the defining features of LLs including their typologies, methodological specificities, stakeholder configurations, expected outcomes, operational challenges, and avenues for further research. Building on this evidence base, the expert focus group contributed to the development of a broader conceptual understanding of LLs. In line with Imenda's (80) definition of a conceptual framework as a structured integration of interrelated concepts, the experts' reflections helped situate the literature within real-world healthcare dynamics and highlighted three overarching considerations: the need to align LL configurations with context-specific socio-technical conditions; the importance of inclusive stakeholder engagement to ensure the relevance and usability of digital health solutions; and the strategic value of integrating LL activities within existing care pathways to support sustainability and scale. These insights informed the interpretive synthesis that led to the formulation of the CLEA framework (Collaborative Living Ecosystem Approach), a conceptual proposal offering actionable strategies to position LLs as integrated, multidimensional platforms capable of addressing complex health and societal needs.

### 5.1 The collaborative living ecosystem approach: core elements

The CLEA framework is articulated around three core elements: (1) a renewed conceptualization of Living Labs, (2) a structured, stakeholder-driven model of collaborative practices, and (3) the integration of LLs within healthcare ecosystems to generate measurable health and societal impact.

First, LLs in digital health are defined as the following:

*LLs are open innovation ecosystems conceived as user-centred networks. In these ecosystems, multiple stakeholders—patients, citizens, caregivers, researchers, healthcare professionals, policymakers, and businesses—collaborate to innovate, develop, and implement new health interventions that encompass innovative health services, therapeutic solutions, and technologies while identifying emerging research opportunities*.

This definition expands on previous works emphasizing the user-centred and co-creation approach (8, 16) by incorporating multidimensional aspects of health. In line with Kim et al. (17), it underscores the necessity for the systemic integration of research and practice to meet evolving healthcare demands. These platforms integrate technologies, practices, and methods to address citizen-centred health and societal needs. Thus, the definition of the CLEA intervention is completed by specifying that:

*LLs interventions integrate technologies, practices, and methods that can be implemented in physical (e.g., healthcare settings, homes, etc.) and virtual environments. LLs simulate or replicate the real-life contexts of users (patients, citizens and communities) within multidisciplinary research areas and settings*.

Second, the CLEA framework offers actionable insights to enhance Living Lab interventions by emphasizing a stakeholder-driven approach. Innovation within LLs often tackles complex, multifaceted challenges, necessitating input from diverse perspectives (45). Unlike previous studies broadly addressing stakeholder participation, the CLEA framework uniquely details these participations. The proposed framework comprehensively classifies stakeholder roles within the LLs ecosystems. Specifically, Table 2 categorizes stakeholders into five distinct groups based on their contributions to key collaborative activities, including co-creation, co-design, co-development, co-testing, and co-evaluation. The *insight contributors* (1), mainly patients, citizens, and vulnerable groups, inform design through lived experiences; the *knowledge specialists* (2), including healthcare professionals and researchers, drive co-design, development, and evaluation with technical and clinical insights; the *developer strategists* (3), such as healthcare providers, developers and researchers, according to their specific competences, refine and test the interventions in real-world contexts, the *innovation enablers* (4), including startups and corporate partners, integrate innovations into systems while ensuring scalability and market readiness, and the *facilitators* (5), such as policymakers and communities, promote ethical alignment and societal acceptance through participatory frameworks. By explicitly identifying and differentiating stakeholder roles based on specific activities—such as co-design or co-development, this framework moves beyond simplistic stakeholder classification to address their practical contributions. It also recognizes that a single stakeholder may transition between roles at different stages of the living lab experience (13). By adopting this dynamic perspective, the proposed framework effectively bridges the gap between theoretical stakeholder engagement and practical implementation in LLs. This approach provides actionable insights for mobilizing diverse expertise throughout the innovation LLs lifecycle.

**Table 2:** Stakeholder roles and collaborative activities.

**Role**	**Typologies of stakeholders**	**Co-creation activities**
Insight Contributors	• **Patients and Caregivers**: provide primary needs and lived experiences that inform design. • **Citizens and Vulnerable Groups:** Provide critical perspectives to ensure inclusivity and accessibility.	• **Co-design:** Share real-life experiences through workshops and discussions, focus groups • **Co-Evaluation**: Offer feedback via usability tests, interviews and surveys.
Knowledge Specialists	• **Healthcare Professionals**: offer clinical expertise and **p**atient-centred insights • **Designers and Developer**s: Provide technical and creative input • **Researchers:** Identify research gaps and align objectives with societal needs.	• **Co-design:** collaborate on iterative prototyping, scenario-based brainstorming, and simulation methods. • **Co-development:** Translate complex concepts into actionable insights. • **Co-Evaluation**: Provide structured, data-driven feedback based on domain expertise.
Developer Strategists	• **Healthcare Providers**: develop research-driven solutions within clinical or operational settings. • **Designers and Developers:** Contribute expertise in knowledge transfer and capacity-building. • **Researchers:** ensure scientific rigour and evidence-based approaches.	• **Co-development:** refine ideas based on technical and operational insights. • **Co-Testing:** Validate solutions in real-world scenarios. • **Co-evaluation**: Analyze effectiveness aligned with knowledge gaps and institutional goals.
Innovation enablers	**Healthcare and other organisations** • Healthcare facilities: ensure organisational integration and implementation. • Research and Educational institutions align solutions with academic and societal goals. **Business partners** • Startup and SMEs: Bring agility, niche innovation and cutting-edge knowledge • Technology Providers: Offer integrative competencies to adopt new technologies and leverage mature innovations for industrialisation. (devices, platforms, etc.) • Corporate Partners: Provide funding, scalability potential, and market insights.	• **Co-development**: ensure solutions integrate seamlessly with the existing system • **Co-Testing:** Implement solutions in operational and real settings. • **Co-evaluation:** Assess market feasibility, usability, scalability and exploitation opportunities.
Facilitators	**Institutions** • Policymakers: Define frameworks and standards that are evidence-based • Government Agencies and Public Authorities: Ensure compliance with regulations and alignment with public interest. **Social groups and their organisations** • Local Communities: Represent societal and cultural values, fostering broader acceptance • Nonprofits and Advocacy Groups: Advocate for equity and ethical considerations in development.	• **Co-design:** develop ethical and institutional frameworks to foster trust-based relationships and promote social acceptance • **Co-evaluation:** Participate in social testing to ensure societal alignment and ethical integrity.

Third, CLEA extends beyond merely a method or a collaborative innovation context; instead, it positions the LLs primarily as an integrative healthcare ecosystem designed to connect and optimize various functionalities. Numerous studies emphasize the diverse outcomes associated with the Living Lab approach, including enhanced healthcare accessibility, improved efficiency, and increased innovation, while also addressing key priorities such as personalized services, training support, and cost reduction (13, 17). To provide a structured analysis of these multisided outcomes, this study categorizes them into three primary domains: methodological advancements, innovation-driven applications, and systemic impact. *Methodological advancements* highlight the role of Living Labs in enhancing research rigor, facilitating co-creation, and ensuring that healthcare research is both scientifically robust and practically applicable (4, 16). By integrating participatory design, real-world testing, and interdisciplinary collaboration, Living Labs bridges the gap between controlled research environments and real-world healthcare challenges, thereby strengthening the evidence base and practical relevance of health interventions. *Innovation-driven applications* leverage cutting-edge technologies, patient-centred digital tools, and data-driven approaches to advance healthcare service provision, enhance patient self-management, and support informed decision-making (17). By optimizing service provision and fostering the integration of preventive and ageing-in-place strategies, Living Labs contribute to the development of more effective and personalized healthcare solutions. *The systemic impact* extends the role of LLs beyond individual interventions, addressing broader structural challenges it encourages by expanding healthcare access, promoting social inclusion, and driving policy and educational growth (12, 43, 58). Through their role in informing regulations and ethical frameworks, supporting workforce training, and fostering sustainable innovation ecosystems, Living Labs contribute to long-term healthcare transformation and equitable service delivery. A concrete example is provided by a recent immersive virtual reality (VR) study for managing dental phobia (81). Clinicians, engineers, and researchers co-designed a VR system tailored to dental procedures, aligning interaction constraints with clinical ergonomics. The solution, tested in a university hospital on odontophobic patients, integrated symbolic interaction to preserve patient immobility during treatment. Real-time testing, combined with physiological monitoring and feedback from both patients and clinicians, enabled iterative refinement (82).

To further structure this categorization, this study builds on the assessment framework proposed by Proctor et al. (81, 82), which offers analytical tools for evaluating the effectiveness, feasibility, and impact of research implementation initiatives across various domains. While previous studies have explored the implementation of Living Lab innovations through Proctor's framework to varying degrees, only a few have considered the full spectrum of potential outcomes (11). Expanding on this approach, the present study introduces overarching indicators that establish a direct connection between implementation outcomes and specific Living Lab outcome domains. This integration provides a more structured and systematic evaluation tool for assessing the impact and effectiveness of Living Lab models. As illustrated in Table 3, the study aligns Proctor's indicators with a categorization of Living Lab outcomes and further proposes concrete, implementable measures tailored to each identified area of interest.

**Table 3:** LL as service integration platforms.

**LL Outcomes Categories**	**Examples of proposed measures (Epm) based on the Proctor et al. (82) framework**
**Methodological Advancements** • Enhance innovation rigour • Facilitate co-creation • Improve research applicability	Acceptability refers to the perceived suitability and satisfaction with a treatment, service, or innovation. • *Epm*: patient and provider satisfaction surveys, qualitative interviews.
	Appropriateness: perceived fit or relevance of the innovation or practice • *Epm*: relevance assessments, expert evaluations, cultural alignment studies.
	Feasibility: successful implementation of an innovation. • *Epm*: resource availability analysis, pilot studies, operational assessments.
	Effectiveness: providing health based on scientific knowledge • *Epm*: clinical trials, outcome-based evaluations, health impact assessments.
**Innovation-Driven Applications** • Improve healthcare services • Strengthen patient self-management • Support data-informed decision	Adoption: initial decision to employ an innovation or practice: • *Epm*: user acceptance rate, percentage of healthcare facilities adopting the innovation, usage analytics.
	Efficiency: avoids waste • *Epm*: reduction in administrative time, resource utilisation metrics, cost-effectiveness analysis.
	Patient-centeredness: respectful and responsive are aligned with patient values • *Epm*: patient satisfaction surveys, adherence to patient preferences in care decisions
	Safety: preventing harm and injuries to primary users • *Epm*: number of reported adverse events, error reduction rates, cybersecurity incident reports.
	Cost: cost impact of the digital innovation • *Epm*: reduction in hospital admission or readmissions, financial burden on patients
	Fidelity: adherence to the original implementation plan of an innovation. • *Epm*: compliance with implementation guidelines, deviation from protocol analysis, adherence to best practices
	Satisfaction: overall health and quality of life • *Epm*: patient-reported outcome measures (PROMs), follow-up survey responses
	Function: ability to function • *Epm*: improved physical and mental health indicators and activity level assessments.
**Systemic impact** • Expand health access • Promote health literacy and social inclusion • Drive policy, education, and economic growth	Equity: quality of health regardless of gender, ethnicity, socio-economic status, or location. • *EPM: Semi-structured interviews and questionnaires on genderism, psychosocial pathways, access to affordable mental and healthcare*, socioeconomic status (education, employment, income), presence of a cultural mediator, and dissemination.
	Penetration: integration of practice/service within a community setting • *Epm*: number of users, adoption rate, improved capabilities, eligible users engaged, service reach index
	Sustainability: the extent to which a newly implemented service/innovation is maintained on time • *Epm*: surveys of communities' satisfaction, Policy or Funding Continuity, long-term changes in key health indicators, affordability Index
	Public health and wellbeing: a measurable change in patient symptoms and citizen health concerns • *EPM: Standardised scales to measure indexes of well-being (anxiety/depression, sleep, nutrition, physical exercise, Positive and Negative Affect, lifestyle, hobbies*, and free time, socialisation).

By consolidating these dimensions, CLEA offers a comprehensive architecture for understanding and operationalizing Living Labs in healthcare. Its added value becomes clear when situated within the broader landscape of existing Living Lab initiatives in Europe. While the framework draws conceptual inspiration from the ENoLL, it advances beyond current implementations by offering a more formalized and evaluative structure for health-innovation ecosystems. For example certified ENoLL Living Labs such as the Healthcare Living Lab Catalonia (83) demonstrate the value of co-creation, prototyping, and real-life validation of digital health technologies or initiatives like the Galician Network of Health Living Labs promote collaborative healthcare solutions across institutional settings (84). However, although these models successfully embody user-centred innovation in real-life contexts, they often lack formalized mechanisms for classifying stakeholder roles, integrating services across the continuum of care, and systematically evaluating implementation outcomes. CLEA addresses these limitations through a structured conceptual framework that promotes integrated service innovation aligned with health system needs and systemic collaboration, defines dynamic stakeholder roles, and links implementation strategies to measurable systemic impacts.

## 6 Conclusion, implications and further research

This study advances the conceptualization of LLs in digital health by introducing the Collaborative Living Ecosystem Approach (CLEA), which reframes LLs as integrated, multidimensional healthcare innovation platforms. This framework is derived from a systematic review and expert insights, providing a comprehensive understanding of the key elements that define LLs. The key contributions of this research are threefold: (1) a refined definition of LLs that emphasizes their role in promoting integrated service innovation grounded in real-world health needs, (2) a stakeholder-driven approach that details the dynamic participation of actors across different co-creation activities, (3) a structured evaluation framework that categorizes LLs outcomes into methodological advancements, innovation-driven applications, and systemic impact (4).

**Figure 2:**
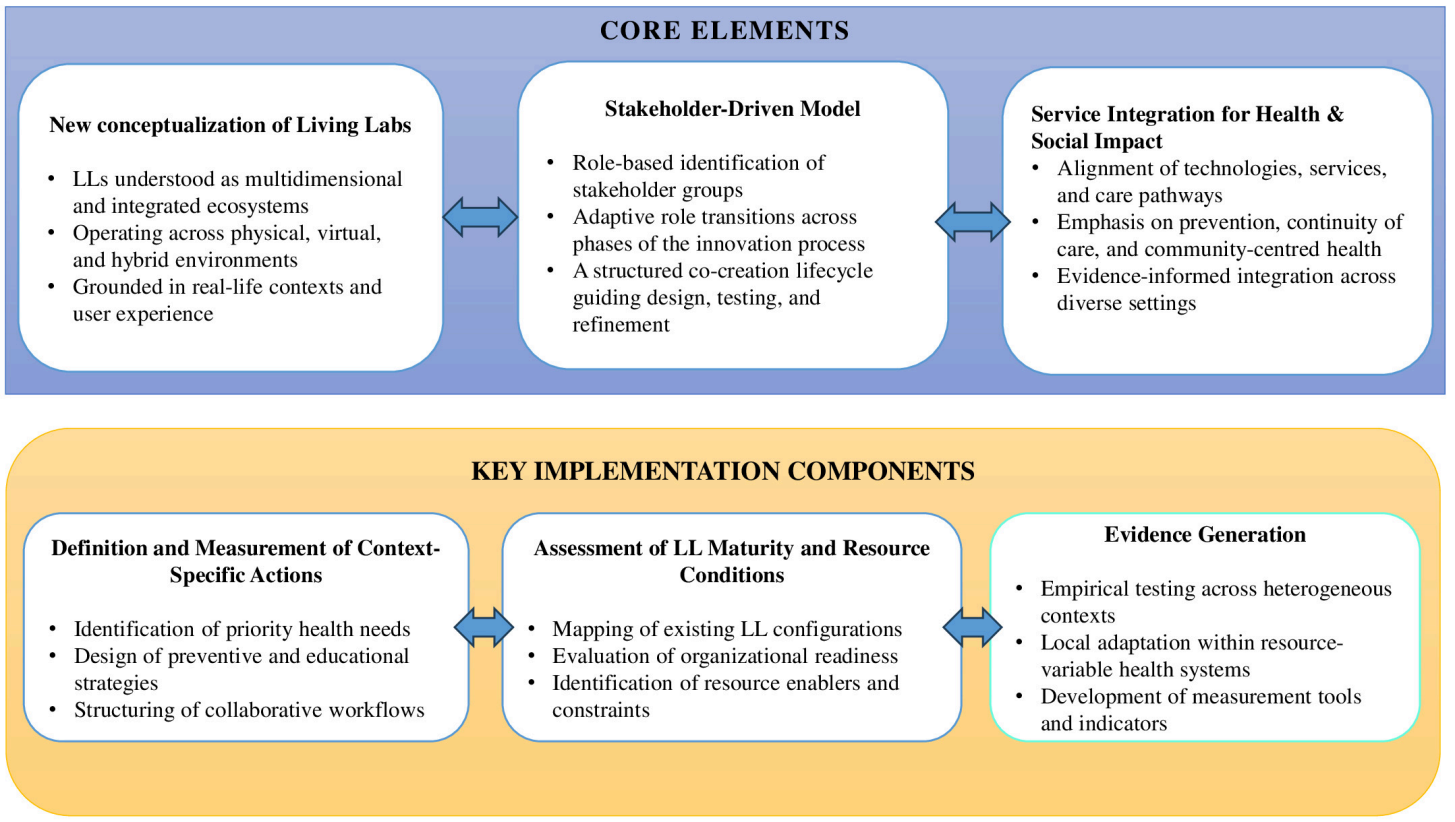
The CLEA framework (collaborative living ecosystem approach).

Figure 2 presents the CLEA framework, structured around two complementary levels. The upper section illustrates its core elements, which serve as conceptual pillars underpinning the framework's theoretical foundations, while the lower section identifies key implementation components that offer a starting point for translating CLEA into real-world applications.

The core elements—reconceptualising LLs as multidimensional environments, structuring multi-stakeholder engagement, and integrating services for health and social impact—serve as a foundation for the broader theoretical implications and future research directions outlined in Table 4. First, the redefined conceptualization of LLs underscores the need for decentralized innovation environments that transcend conventional methodological frameworks and spatially confined experimental settings. LLs should be understood as dynamic, collaborative ecosystems that foster the continuous co-development of healthcare technologies, practices, and interventions (4, 13). This shift has significant implications for healthcare practitioners, underscoring the importance of multidisciplinary competencies that encompass health, research, and health innovation. Universities, higher education institutions, and research centres could benefit from the collaboration in the LLs' experience to create new research opportunities. They offer new pathways for identifying and addressing emerging population health needs, particularly in the area of prevention. They also establish valuable avenues for early-career researchers to develop, refine, and apply their skills in a real-world setting. By positioning LLs as experimental learning ecosystems, healthcare practitioners can foster capacity-building, interdisciplinary collaboration, and evidence-based interventions to optimize care and prevention throughout the life cycle. In addition, given the rapid evolution of emerging technologies—including artificial intelligence, robotics, chatbots, and serious games—future research should explore the potential of hybrid context more systematically and how it could expand the accessibility and scalability of LLs initiatives while maintaining real-world applicability. Thus, investing in LLs as long-term infrastructures within healthcare systems, rather than temporary innovation projects, can also strengthen health resilience, promote health transformation, and encourage cross-sector partnerships in public health. For policymakers, this redefinition underscores the importance of positioning LLs as service provision mechanisms for innovation, advocating for investment in digital transformation, cross-sector partnerships, and integration into healthcare ecosystem practices. The CLEA framework can support a scalability model, ensuring that LL can be equally implemented in health service-deprived areas with minimal technological requirements as long as they remain connected to a broader community to provide capacity building. Thus, further research should explore how to effectively implement and scale decentralized LLs models to ensure they remain adaptable to evolving healthcare needs.

**Table 4:** Implications and further research.

**Contribution**	**Connection to the literature**	**Implications**	**Further research**
LLs as Collaborative Innovation Ecosystems	• Decentralised Innovation Environments that function as open innovation environments, rather than spatially confined experimental sites (2, 4, 9, 16). • Continuous and adaptive ecosystems that evolve iteratively in response to real-world healthcare challenges rather than relying on controlled, single-goal interventions (10, 12, 13).	**For Practitioners:** Supporting decentralised innovation requires multidisciplinary expertise in healthcare and research, enhancing collaboration between universities, research centers, and healthcare professionals for more effective prevention and treatment strategies. **For Policymakers**: Positioning LLs as long-term infrastructures for innovation, urging investment in digital transformation, cross-sector partnerships, and health system resilience.	What models best integrate long-term learning (LLs) into healthcare for effective treatment? How can hybrid (physical-digital) LLs improve accessibility and real-world impact? – What policy incentives drive cross-sector collaboration in digital health?
Multi-stakeholder engagement for Sustainable Co-Creation	• Categorisation of stakeholder roles based on their contributions to key innovation activities (4, 16). • Evolving roles of stakeholders and the multi-sided nature of their engagement (2, 7, 10).	**For Practitioners:** promoting a structured governance model (e.g., hub-and-spoke) that is multi-actor, multi-context, and fosters co-creation among multiple stakeholders. **For policymakers**, encouraging early involvement of regulators, public authorities, and communities will help establish evidence-based ethical and institutional frameworks.	What governance models best strike a balance between central coordination and stakeholder-driven innovation? How can early regulatory involvement enhance the effectiveness and compliance of local laws (LLs)? What mechanisms ensure that ethical and institutional standards evolve in tandem with technological advancements?
Service integration platform for Scalable and Impactful Healthcare Solutions	• LLs are structured innovation ecosystems bridging the gap between research advancements and real-world healthcare applications (12, 13, 17). • Value-based healthcare models leveraging LL-generated solutions to enhance patient-centred care and citizen health (58).	**For Practitioners:** integrating LL frameworks to facilitate seamless transitions between experimental innovation and healthcare implementation. **For Policymakers:** Encouraging policies that foster scalability and interoperability of LL-driven innovations.	How can LLs enhance the integration of digital health innovations into mainstream healthcare services? What metrics best capture the effectiveness of LL-driven innovations in value-based healthcare? How can LLs contribute to the development of sustainable, scalable, and interoperable digital health solutions?

Second, the multiple stakeholder-driven approach the model proposes, while providing evidence of stakeholder participation according to dynamic and evolving roles, raises several challenges for practitioners, particularly regarding the coordination and engagement of diverse actors. The complexity of managing multiple stakeholders underscores the need for structured governance mechanisms that facilitate sustained collaboration and efficient resource management. In response to these challenges, governance models such as the hub-and-spoke framework could offer a structured yet flexible approach to organizing LLs. The broader distribution of LLs to address community-driven health needs presents the challenge of ensuring sustainability with minimal equipment within a highly decentralized model. This aspect necessitates further research into governance mechanisms that support a hub-and-spoke model, striking a balance between centralized coordination and decentralized innovation. Such a model would enhance the adaptability of LLs to technological advancements and the evolving needs of diverse populations.

Additionally, the potential integration of low-tech LLs into human-intensive environments—such as schools, religious institutions, and older adult centres—warrants further research exploration to maximize accessibility and community engagement. Beyond governance structures, the effectiveness of LLs also depends on proactive policymaking. Engaging regulatory bodies from the early stages of LLs development ensures alignment with public health priorities and legal frameworks, facilitating smoother implementation and mitigating compliance risks. Expanding stakeholder networks to include community organizations, patient groups, and caregivers can enhance user engagement, improve treatment adherence, and provide essential psychological and social support. Encouraging broader participation strengthens the societal relevance of LLs, ensuring that digital health innovations are effectively integrated into users' daily lives. Future studies could investigate the best practices for institutionalizing early regulatory involvement and fostering long-term stakeholder engagement and community participation.

Third, CLEA's structured approach to categorizing LLs outcomes strengthens the understanding of LLs as service platforms, offering insights into their long-term effectiveness and adaptability in digital health interventions. By examining how different LL models integrate research advancements with real-world applicability, practitioners can adopt a holistic framework that captures their methodological, technological, and systemic contributions. This perspective highlights the complexity of LLs—not just as experimental environments but as structured innovation ecosystems that support iterative learning, co-creation, and the seamless translation of novel solutions into practical healthcare applications. A key challenge remains to ensure that LLs-generated solutions are successfully embedded into broader healthcare pathways. Innovations must transition from experimental settings to mainstream applications while remaining capable of monitoring and adapting to evolving needs, stakeholder roles, and healthcare contexts. Considering these aspects, policymakers should prioritize institutionalizing robust outcome measurement frameworks that drive LLs improvements in healthcare ecosystems. Moreover, while existing frameworks provide valuable analytical tools, assessment metrics must be refined to better capture the dynamic interactions between innovation, policy, and societal adoption. An important area of inquiry is the role of LLs in value-based healthcare models—particularly in determining whether LL-driven innovations generate measurable benefits at both the patient and system levels. Additionally, empirical studies are needed to evaluate the scalability and interoperability of LL solutions, especially their integration into existing healthcare infrastructures.

Finally, given the conceptual foundations of this study, an essential research trajectory involves the exploration of key implementation components, which offer a starting point for translating the CLEA framework into practice. A first step in this pathway involves assessing how the framework can inform the definition of context-specific actions—such as the activation of local preventive and educational strategies, and the design of collaborative workflows suited to diverse implementation environments. In parallel, the validation process should examine the maturity and readiness of existing Living Lab ecosystems, through activities such as mapping LL configurations, evaluating organizational preparedness, and identifying enablers and barriers to implementation. Given the importance of continuous learning and evidence loops, empirical research across heterogeneous healthcare contexts will be necessary to test the framework's robustness and adaptability to different organisational cultures, or governance structures. Such studies will also support the development of context-sensitive indicators and measurement tools. Understanding how CLEA operates within such diverse settings will be essential for assessing its applicability and long-term relevance.
