# Supplementary material for: Living Labs in digital health: a collaborative ecosystem approach for continuum of care
Source: Front Public Health. 2026 Jan 6;13:1728904. doi: 10.3389/fpubh.2025.1728904 (PMC12816325; doi:10.3389/fpubh.2025.1728904)
Supplement: Supplementary file 1 [file Supplementary_file_1.docx]

**Supplementary Table 1: LLs Main definitions**

| **Author(s)** |  | **Open innovation (eco)system** | **User-centred** | **Network innovation** | **LL Environment** | **LL Environment** |
| --- | --- | --- | --- | --- | --- | --- |
|  |  |  |  |  | **Physical** | **Virtual** |
| **Aharony et al., 2011** |  |  |  | ✓ |  |  |
| **Almeida et al., 2022** |  |  | ✓ |  |  | ✓ |
| **Brauner et al., 2015** |  |  |  |  | ✓ |  |
| **Byrne et al., 2023** |  | ✓ |  |  |  |  |
| **Callari et al., 2020** |  |  | ✓ |  |  |  |
| **Callari et al., 2019** |  |  | ✓ |  |  |  |
| **Cuomo et al., 2021** |  |  |  |  |  |  |
| **De Witte et al., 2021** |  | ✓ |  |  | ✓ |  |
| **Demiris et al., 2013** |  |  |  | ✓ |  |  |
| **Dietrich et al., 2021** |  | ✓ |  |  | ✓ |  |
| **Fleet et al., 2020** |  | ✓ |  |  |  |  |
| **Fotis et al., 2023** |  | ✓ | ✓ |  |  | ✓ |
| **Georges et al., 2016** |  | ✓ | ✓ |  |  |  |
| **Hamm et al., 2019** |  |  |  |  | ✓ |  |
| **Haukipuro et al., 2019** |  |  | ✓ |  |  | ✓ |
| **Hogervorst et al., 2023** |  | ✓ | ✓ |  |  |  |
| **Kanstrup et al., 2010** |  | ✓ |  |  |  |  |
| **Kim et al., 2020** |  |  | ✓ |  |  |  |
| **Kim and Choi, 2023** |  | ✓ |  |  |  |  |
| **Kwon and Ju, 2023** |  | ✓ | ✓ |  |  |  |
| **Leminen et al., 2017** |  | ✓ |  | ✓ |  | ✓ |
| **Lepik and Krigul 2021** |  | ✓ |  |  | ✓ |  |
| **Lupton et al 2017** |  | ✓ |  |  |  |  |
| **Mariano et al., 2023** |  |  |  |  | ✓ |  |
| **Martinez et al., 2016** |  |  | ✓ |  | ✓ |  |
| **Merino-Barbancho et al., 2023** |  |  | ✓ |  |  |  |
| **Montalva Colomer et al., 2014** |  |  |  |  | ✓ |  |
| **Morand et al., 2023** |  |  | ✓ | ✓ |  |  |
| **Palmer et al., 2023** |  | ✓ | ✓ |  |  |  |
| **Samuelson et al., 2023** |  | ✓ |  |  |  | ✓ |
| **Schmitz et al., 2022** |  | ✓ | ✓ |  |  |  |
| **Snaphaan et al., 2022** |  |  | ✓ |  |  |  |
| **Spinelli et al., 2019** |  | ✓ |  |  |  |  |
| **Swinkels et al., 2018** |  | ✓ | ✓ |  |  |  |
| **Ward et al., 2015** |  |  | ✓ |  |  |  |

**Supplementary Table 2: LLs Contexts/ Fields of use**

| **Author(s)** | **Primary Care** | **Chronic Diseases** | **Emergency Medical Situations** | **Prevention** | **Psychological Well-being & Mental Health Disease** | **Others** |
| --- | --- | --- | --- | --- | --- | --- |
| **Aharony et al., 2011** | ✔ |  |  |  | ✔ (Psychological well-being) |  |
| **Aguirre et al., 2016** | ✔ | ✔ (Generic or other chronic disease) |  |  |  |  |
| **Almeida et al., 2022** |  | ✔ (Vulnerable elders) |  |  |  |  |
| **Benvenuto et al., 2017** | ✔ | ✔ (Heart failure) |  |  |  |  |
| **Brauner et al., 2015** | ✔ |  |  |  |  |  |
| **Burrows et al., 2022** |  |  |  |  | ✔ (Psychological well-being) |  |
| **Busnatu et al., 2022** |  | ✔ (Cardiac disease) |  |  |  |  |
| **Byrne et al., 2023** |  | ✔ (Respiratory disease) |  |  |  |  |
| **Callari et al., 2020** |  |  |  |  |  | ✔ |
| **Callari et al., 2019** |  | ✔ (Aging population) |  |  |  |  |
| **Cuomo et al., 2021** | ✔ |  |  |  |  |  |
| **De Witte et al., 2021** | ✔ |  |  |  |  |  |
| **Demiris et al., 2013** | ✔ |  |  |  |  |  |
| **Dietrich et al., 2021** |  |  |  | ✔ (Alcohol prevention) |  |  |
| **Fleet et al., 2020** |  |  | ✔ (Emergency services in remote areas) |  |  |  |
| **Fotis et al., 2023** |  |  |  |  |  |  |
| **Georges et al., 2016** |  |  |  |  |  | ✔ |
| **Hamm et al., 2019** |  |  |  | ✔ (Fall risk prevention) |  |  |
| **Harvey and Jones 2022** |  |  | ✔ (Emergency management in rural healthcare) |  |  |  |
| **Haukipuro et al., 2019** |  |  |  |  |  | ✔ |
| **Haux et al., 2014** |  | ✔ |  |  |  |  |
| **Hogervorst et al., 2023** |  | ✔ |  |  |  |  |
| **Kanstrup, 2017** |  |  |  |  | (Psychological well-being) |  |
| **Kanstrup, 2014** |  | ✔ (Diabetes) |  |  |  |  |
| **Kanstrup et al., 2010** |  | ✔ (Diabetes) |  |  |  |  |
| **Kim et al., 2020** | ✔ |  |  |  |  |  |
| **Kim et al., 2021** | ✔ |  |  |  |  |  |
| **Kim et al., 2022** |  | ✔ (Cardiovascular |  |  |  |  |
| **Kim and Choi, 2023** |  |  |  |  | ✔ (Mental health disease) |  |
| **Kwon and Ju, 2023** | ✔ |  |  |  |  |  |
| **Leminen et al., 2017** |  |  |  |  |  | ✔ |
| **Lepik and Krigul 2021** |  |  |  |  |  | ✔ |
| **Lindskrog et al., 2019** |  | ✔ (COPD) |  |  |  |  |
| **Lobo et al., 2022** |  | ✔ (Cardiac disease) |  |  |  |  |
| **Luengo-Polo et al., 2021** | ✔ |  |  |  |  |  |
| **Lupton er al 2017** |  | ✔ |  |  |  |  |
| **Mariano et al., 2023** |  |  |  |  |  | ✔ |
| **Martinez et al., 2016** | ✔ |  |  |  |  |  |
| **Merino-Barbancho et al., 2023** | ✔ |  |  |  |  |  |
| **Montalva Colomer et al., 2014** |  | ✔ (Generic or other chronic disease) |  |  |  |  |
| **Morand et al., 2023** |  |  | ✔ (OHCA/FBAO) |  |  |  |
| **Noublanche et al., 2019** | ✔ |  |  |  |  |  |
| **Palmer et al., 2023** |  |  |  |  | ✔ (Mental health disease) |  |
| **Phanareth et al., 2017** |  | ✔ (COPD) |  |  |  |  |
| **Rahman et al., 2022** | ✔ |  |  |  |  |  |
| **Rumeau et al., 2021** | ✔ |  |  |  |  |  |
| **Samuelson et al., 2023** | ✔ |  |  | ✔ (Fall risk prevention) |  |  |
| **Santa et al., 2023** |  |  |  |  | ✔ (Psychological well-being) |  |
| **Schmitz et al., 2022** |  | ✔ (Cardiac disease) |  |  |  |  |
| **Seo et al., 2020** |  |  |  |  |  | ✔ |
| **Snaphaan et al., 2022** |  |  |  |  | ✔ (Mental health disease) |  |
| **Spinelli et al., 2019** | ✔ |  |  |  |  |  |
| **Swinkels et al., 2018** | ✔ | ✔ (COPD, Cardiac disease, Diabetes) |  |  |  |  |
| **Vaziri et al., 2016** |  |  |  | ✔ (Fall risk prevention) |  |  |
| **Verbeek et al., 2020** | ✔ |  |  |  |  |  |
| **Ward et al., 2015** |  |  |  |  | ✔ (Mental health disease) |  |

**Supplementary Table 3: LLs Health-related innovations**

| **Author(s)** | **Apps/ Serious Games/Exercise Games** | **Software/Platforms** | **Wearables/Sensors/Robots/Chatbot** | **new Protocol/procedure** | **Others** |
| --- | --- | --- | --- | --- | --- |
| **Aharony et al., 2011** |  |  | Mobile sensors for behavioural monitoring |  |  |
| **Aguirre et al., 2016** |  | Context-Aware e-Health system integrating social and health sensors |  |  |  |
| **Almeida et al., 2022** |  |  |  | Protocol for managing older adults in healthcare (Equity and Quality Checklist) |  |
| **Benvenuto et al., 2017** |  |  |  | Management, evaluation, and treatment method for older patients |  |
| **Brauner et al., 2015** | Fitness farm" serious exercise game |  |  |  |  |
| **Burrows et al., 2022** | Emotional support app for the homeless |  |  |  |  |
| **Busnatu et al., 2022** |  |  | Virtual assistant for remote cardiac rehabilitation |  |  |
| **Byrne et al., 2023** |  | Digital Health Technology for Respiratory Patients​ |  |  |  |
| **Callari et al., 2020** |  |  |  | Ethical framework for healthcare research in Living Labs |  |
| **Callari et al., 2019** |  |  |  |  | ✔ |
| **Cuomo et al., 2021** |  |  |  |  | ✔ |
| **De Witte et al., 2021** |  |  |  |  | ✔ |
| **Demiris et al., 2013** |  | Platform visualizing older adults' wellness |  |  |  |
| **Dietrich et al., 2021** | Virtual reality simulation for alcohol prevention |  |  |  |  |
| **Fleet et al., 2020** |  | Telemedicine Platform (Reacts) for Remote Emergency Support​ |  |  |  |
| **Fotis et al., 2023** |  |  |  |  | ✔ |
| **Georges et al., 2016** |  |  |  | Guidelines to reduce test user drop-outs in LL field trials |  |
| **Hamm et al., 2019** | Fall risk prevention app |  |  |  |  |
| **Harvey and Jones 2022** |  |  |  |  | ✔ |
| **Haukipuro et al., 2019** |  |  |  | Recruitment/testing methodology through citizen-institution collaboration |  |
| **Haux et al., 2014** |  | PAHA system for human-machine interaction paradigms |  |  |  |
| **Hogervorst et al., 2023** |  |  |  | Methodology to improve medication adherence after interventions |  |
| **Kanstrup, 2017** |  |  |  |  | ✔ |
| **Kanstrup, 2014** |  |  |  |  | ✔ |
| **Kanstrup et al., 2010** |  | Platform to assist people with diabetes and their relatives |  |  |  |
| **Kim et al., 2020** | Health app for Korean-Chinese working women |  |  |  |  |
| **Kim et al., 2021** |  |  |  |  | New health engagement program to enhance self-care agency among migrant workers​. |
| **Kim et al., 2022** | Mobile app-based walking |  |  |  |  |
| **Kim and Choi, 2023** | Mental health app |  |  |  |  |
| **Kwon and Ju, 2023** |  |  | Companion robot |  |  |
| **Leminen et al., 2017** |  |  | Service robot | Framework for analysing service innovations enabled by robotics; Methodology of collaboration between technology designers and users in care homes |  |
| **Lepik and Krigul 2021** |  |  |  |  | ✔ |
| **Lindskrog et al., 2019** |  | Algorithm for self-monitoring |  |  |  |
| **Lobo et al., 2022** | Stroke patient support app |  |  |  |  |
| **Luengo-Polo et al., 2021** |  |  | Robot supporting older adults |  |  |
| **Lupton er al 2017** |  | Integration of apps, platforms, wearables, and telemedicine for better healthcare management​. |  |  |  |
| **Mariano et al., 2023** |  |  |  |  | ✔ |
| **Martinez et al., 2016** |  | Telemedicine for home-based patient monitoring |  |  |  |
| **Merino-Barbancho et al., 2023** |  |  |  | MAHA (Madrid Active and Healthy Ageing) CLUB |  |
| **Montalva Colomer et al., 2014** |  |  | Smart house equipped with ICT devices |  |  |
| **Morand et al., 2023** |  |  |  | Emergency procedures to guide bystanders |  |
| **Noublanche et al., 2019** |  |  | Equipped hospital room with sensors for vital signs and movement tracking |  |  |
| **Palmer et al., 2023** |  |  |  | "Togetherness-by-Design" framework for participatory design addressing injustices |  |
| **Phanareth et al., 2017** |  |  |  | Capital Care Model (ECM) integrating digital tech for chronic disease management |  |
| **Rahman et al., 2022** |  |  | Biomarkers measurement wearable |  |  |
| **Rumeau et al., 2021** |  |  |  |  | ✔ |
| **Samuelson et al., 2023** |  |  |  |  | ✔ |
| **Santa et al., 2023** |  | Platform for mental health support |  |  |  |
| **Schmitz et al., 2022** |  | TIMELY health platform for cardiac rehabilitation patients |  |  |  |
| **Seo et al., 2020** |  |  |  |  | ✔ |
| **Snaphaan et al., 2022** | Serious game for dementia patients |  |  |  |  |
| **Spinelli et al., 2019** |  |  |  |  | ✔ |
| **Swinkels et al., 2018** | Online video consultation for COPD patients |  |  |  |  |
| **Vaziri et al., 2016** |  | ICT-based system iStoppFalls |  |  |  |
| **Verbeek et al., 2020** |  |  |  |  | ✔ |
| **Ward et al., 2015** |  | AroundMe™ service for monitoring older adults’ vital signs |  |  |  |

**Supplementary Table 4: Stakeholders main categories**

| **Author(s)** | **Primary Users** | | | | **Healthcare professionals and Design Professionals** | | | **Industries and private partners** | | **Organisations and other Institutions** | | | **Policymakers and Authorities** | | **Communities and Societies** |
| --- | --- | --- | --- | --- | --- | --- | --- | --- | --- | --- | --- | --- | --- | --- | --- |
|  | **Patient (almost all)** | **General Users** | **Caregivers and Families** | **Vulnerable Groups** | **Healthcare Providers** | **Developers** | **Researchers** | **Technology Providers:** | **SMEs and start-up partners:** | **Health care facilities** | **Educational Institutions** | **Research Canters** | **Policymaker** | **Government and Authorities** | **Communities/citizen** |
| Aharony et al., 2011 | ✔ |  |  |  |  |  |  |  |  |  |  |  |  | ✔ |  |
| Aguirre et al., 2016 | ✔ |  |  |  |  |  |  |  |  |  |  |  |  |  | ✔ |
| Almeida et al., 2022 | ✔ |  |  | ✔ |  |  |  |  |  | ✔ |  |  |  |  |  |
| Benvenuto et al., 2017 | ✔ |  |  |  |  |  |  |  |  | ✔ |  |  |  |  |  |
| Brauner et al., 2015 | ✔ | ✔ |  |  |  |  |  |  |  |  |  |  |  |  |  |
| Burrows et al., 2022 | ✔ |  |  | ✔ |  |  |  |  |  |  |  |  |  |  |  |
| Busnatu et al., 2022 | ✔ | ✔ |  |  | ✔ |  |  |  |  |  |  |  |  |  |  |
| Byrne et al., 2023 | ✔ |  |  |  |  |  |  |  |  |  |  | ✔ |  |  |  |
| Callari et al., 2020 | ✔ |  |  |  |  |  |  |  |  | ✔ |  |  |  |  |  |
| Callari et al., 2019 | ✔ |  | ✔ |  |  |  |  |  |  |  |  |  |  |  |  |
| Cuomo et al., 2021 | ✔ |  |  |  |  |  |  |  |  |  |  |  |  |  |  |
| De Witte et al., 2021 | ✔ |  |  |  |  |  |  |  |  |  | ✔ |  |  |  |  |
| Demiris et al., 2013 | ✔ |  |  |  | ✔ |  |  |  |  |  |  |  |  |  | ✔ |
| Dietrich et al., 2021 | ✔ |  |  |  |  |  |  |  | ✔ |  |  |  |  |  |  |
| Fleet et al., 2020 | ✔ |  |  |  |  |  |  |  |  |  |  |  |  |  | ✔ |
| Fotis et al., 2023 | ✔ |  |  |  |  |  | ✔ |  |  |  |  |  |  |  |  |
| Georges et al., 2016 | ✔ |  |  |  |  |  |  |  |  |  |  |  |  |  |  |
| Hamm et al., 2019 | ✔ |  |  |  |  | ✔ |  |  |  |  |  |  |  |  |  |
| Harvey and Jones, 2022 | ✔ |  |  |  | ✔ |  |  |  |  |  |  |  |  |  |  |
| Haukipuro et al., 2019 | ✔ |  |  |  |  |  |  |  |  |  | ✔ |  |  |  |  |
| Haux et al., 2014 | ✔ |  |  |  |  |  |  |  |  |  |  |  |  |  |  |
| Hogervorst et al., 2023 | ✔ | ✔ |  |  |  |  |  |  |  |  |  |  |  |  |  |
| Kanstrup, 2017 | ✔ |  |  |  |  |  |  |  |  |  |  |  |  |  |  |
| Kanstrup, 2014 | ✔ | ✔ |  |  |  |  |  |  |  |  |  |  |  |  |  |
| Kanstrup et al., 2010 | ✔ |  |  |  |  |  | ✓ |  |  |  |  |  |  |  |  |
| Kim et al., 2020 | ✔ |  |  |  |  |  |  |  |  |  |  |  |  |  |  |
| Kim et al., 2021 | ✔ |  |  |  |  |  |  |  |  |  |  |  |  |  | ✓ |
| Kim et al., 2022 | ✔ |  |  | ✓ |  |  |  |  |  |  |  |  |  |  |  |
| Kim and Choi, 2023 | ✔ |  |  |  |  |  |  |  |  |  |  |  |  |  |  |
| Kwon and Ju, 2023 | ✔ |  |  |  |  | ✓ |  |  |  |  |  |  |  |  |  |
| Leminen et al., 2017 | ✔ |  |  |  |  |  |  | ✔ |  |  |  |  |  | ✓ |  |
| Lepik and Krigul, 2021 | ✔ |  |  |  |  |  |  |  | ✔ |  |  |  |  |  |  |
| Lindskrog et al., 2019 | ✔ |  |  |  |  |  |  |  |  |  |  |  | ✓ |  |  |
| Lobo et al., 2022 | ✔ | ✓ |  |  |  |  |  |  |  |  |  |  |  |  |  |
| Luengo-Polo et al., 2021 | ✔ |  |  |  |  |  |  |  |  |  |  |  |  |  |  |
| Lupton et al., 2017 | ✔ |  |  |  |  | ✓ |  |  |  |  |  |  |  |  |  |
| Mariano et al., 2023 | ✔ |  |  |  |  |  |  |  |  |  |  |  | ✓ |  |  |
| Martinez et al., 2016 | ✔ |  |  |  |  |  |  |  |  |  |  | ✓ |  | ✓ |  |
| Merino-Barbancho et al., 2023 | ✔ |  |  |  |  |  |  |  |  |  |  |  |  |  |  |
| Montalva Colomer et al., 2014 | ✔ |  |  |  |  |  |  |  |  |  |  |  |  |  |  |
| Morand et al., 2023 | ✔ |  |  | ✔ |  |  |  |  |  |  |  |  |  |  |  |
| Noublanche et al., 2019 | ✔ |  |  |  |  |  |  |  |  | ✓ |  |  |  |  |  |
| Palmer et al., 2023 | ✔ |  | ✓ |  |  |  |  |  |  |  |  |  |  |  |  |
| Phanareth et al., 2017 | ✔ |  |  |  |  |  |  |  |  |  |  |  | ✓ |  |  |
| Rahman et al., 2022 | ✔ |  |  |  |  |  |  |  |  |  |  |  |  |  |  |
| Rumeau et al., 2021 | ✔ |  |  |  |  |  |  |  |  |  |  |  |  |  |  |
| Samuelson et al., 2023 | ✔ | ✓ |  |  |  |  |  |  |  |  |  |  |  |  |  |
| Santa et al., 2023 | ✔ |  |  |  |  |  | ✓ |  |  |  |  |  |  |  |  |
| Schmitz et al., 2022 | ✔ | ✓ |  |  |  |  |  |  |  |  |  |  |  |  |  |
| Seo et al., 2020 | ✔ |  |  |  |  |  |  | ✔ |  |  |  |  |  |  |  |
| Snaphaan et al., 2022 | ✔ | ✓ |  |  |  |  |  |  |  |  |  |  |  |  |  |
| Spinelli et al., 2019 | ✔ |  | ✓ |  |  |  |  |  |  |  |  |  |  |  |  |
| Swinkels et al., 2018 | ✔ |  |  |  |  |  |  |  |  |  |  |  |  |  |  |
| Vaziri et al., 2016 | ✔ |  |  |  | ✓ |  |  |  |  |  |  |  |  |  |  |
| Verbeek et al., 2020 | ✔ |  |  |  |  | ✓ |  |  |  |  |  |  |  |  |  |
| Ward et al., 2015 | ✔ |  |  |  |  |  |  |  |  |  |  |  |  |  |  |

**Supplementary Table 5: Co-creation activities**

| **Author(s)** | **Co-design** | | | **Co-development** | | | | **Co-testing** | | | **Co-Evaluation** | | |
| --- | --- | --- | --- | --- | --- | --- | --- | --- | --- | --- | --- | --- | --- |
|  | **Collaborative workshops** | **Stakeholder problem identification mapping** | **Open discussions and brainstorming** | **Iterative prototyping** | **Participatory Service design** | **Scenario-based methods** | **Mock-up design and participatory implementation program** | **Real-world testing** | **Real life simulation** | **Behavioural observation** | **User assessment** | **Performance metrics** | **Risk assessment** |
| Aharony et al., 2011 |  |  | ✓ |  |  |  |  |  |  |  |  |  |  |
| Aguirre et al., 2016 | ✓ |  |  |  |  |  | ✓ |  |  |  |  |  |  |
| Benvenuto et al., 2017 |  |  | ✓ |  |  |  |  |  |  |  |  |  |  |
| Brauner et al., 2015 | ✓ |  |  |  |  |  |  |  |  |  |  |  |  |
| Burrows et al., 2022 |  | ✓ |  |  |  |  |  | ✓ |  |  |  | ✓ | ✓ |
| Busnatu et al., 2022 |  |  |  | ✓ |  |  |  |  |  |  | ✓ |  |  |
| Callari et al., 2020 | ✓ |  |  |  |  |  |  |  |  |  |  |  | ✓ |
| Callari et al., 2019 |  |  | ✓ |  |  |  |  |  |  |  |  |  |  |
| Cuomo et al., 2021 |  |  |  |  |  | ✓ |  |  |  |  |  | ✓ |  |
| Dietrich et al., 2021 |  |  |  | ✓ |  |  |  | ✓ |  |  |  |  |  |
| Fleet et al., 2020 | ✓ |  |  |  | ✓ |  |  |  |  |  |  | ✓ |  |
| Georges et al., 2016 |  |  |  |  |  |  |  | ✓ |  |  |  |  | ✓ |
| Hamm et al., 2019 |  |  |  |  |  |  |  |  |  |  |  |  |  |
| Harvey and Jones, 2022 |  |  |  |  | ✓ | ✓ | ✓ |  |  |  | ✓ |  |  |
| Haukipuro et al., 2019 |  |  |  |  |  |  |  | ✓ |  |  |  | ✓ |  |
| Haux et al., 2014 |  |  |  | ✓ |  |  |  |  | ✓ |  |  |  |  |
| Hogervorst et al., 2023 | ✓ |  |  |  |  |  | ✓ |  |  | ✓ |  |  |  |
| Kanstrup, 2014 |  | ✓ |  |  |  |  | ✓ |  |  |  |  |  |  |
| Kanstrup et al., 2010 |  |  |  |  |  |  |  |  |  | ✓ |  |  |  |
| Kim et al., 2020 | ✓ |  |  | ✓ |  |  |  |  |  |  | ✓ |  |  |
| Kim et al., 2021 |  | ✓ |  |  |  |  | ✓ |  |  |  |  |  |  |
| Kim et al., 2022 |  | ✓ |  |  |  |  |  | ✓ |  |  |  | ✓ | ✓ |
| Kwon and Ju, 2023 |  |  |  |  |  | ✓ |  |  |  |  |  |  |  |
| Leminen et al., 2017 |  |  |  |  | ✓ |  |  |  |  |  |  |  |  |
| Lepik and Krigul, 2021 |  |  |  |  |  |  |  | ✓ |  |  |  |  |  |
| Lobo et al., 2022 |  |  |  | ✓ |  |  |  |  |  |  | ✓ |  |  |
| Lupton et al., 2017 | ✓ |  |  |  |  |  |  |  |  |  |  |  |  |
| Mariano et al., 2023 |  | ✓ |  |  |  |  |  |  |  |  |  |  |  |
| Martinez et al., 2016 |  |  |  |  |  |  |  |  | ✓ |  |  |  |  |
| Montalva Colomer et al., 2014 |  |  |  |  |  |  |  | ✓ |  |  |  |  |  |
| Morand et al., 2023 |  |  |  |  |  |  |  |  |  |  |  | ✓ | ✓ |
| Noublanche et al., 2019 |  |  |  |  |  |  |  |  |  |  |  | ✓ |  |
| Palmer et al., 2023 |  |  |  |  |  |  |  | ✓ |  | ✓ |  |  |  |
| Phanareth et al., 2017 |  |  |  |  | ✓ |  |  |  |  |  |  |  |  |
| Rahman et al., 2022 |  |  |  |  |  |  |  | ✓ |  |  |  |  | ✓ |
| Rumeau et al., 2021 | ✓ |  |  |  |  | ✓ |  |  |  |  |  |  |  |
| Santa et al., 2023 |  |  |  | ✓ |  |  |  |  | ✓ | ✓ |  |  |  |
| Schmitz et al., 2022 |  |  |  |  |  |  |  |  |  |  | ✓ |  |  |
| Swinkels et al., 2018 | ✓ |  |  | ✓ | ✓ |  | ✓ |  | ✓ |  |  |  |  |
| Vaziri et al., 2016 |  |  |  |  |  |  |  |  |  |  |  |  |  |
| Verbeek et al., 2020 |  |  |  | ✓ | ✓ |  |  |  |  |  |  |  |  |

**Supplementary Table 6: LLs innovation phases**

| **Author(s)** | **Development** | **Implementation** | **User Practices** |
| --- | --- | --- | --- |
| Aharony et al., 2011 |  | ✓ |  |
| Aguirre et al., 2016 |  | ✓ |  |
| Almeida et al., 2022 |  |  |  |
| Benvenuto et al., 2017 |  |  | ✓ |
| Brauner et al., 2015 |  | ✓ |  |
| Burrows et al., 2022 |  |  | ✓ |
| Busnatu et al., 2022 |  | ✓ |  |
| Byrne et al., 2023 | ✓ |  |  |
| Demiris et al., 2013 |  | ✓ |  |
| Georges et al., 2016 | ✓ |  |  |
| Hamm et al., 2019 |  |  | ✓ |
| Harvey and Jones, 2022 | ✓ |  |  |
| Haukipuro et al., 2019 |  | ✓ |  |
| Haux et al., 2014 | ✓ |  |  |
| Hogervorst et al., 2023 |  |  | ✓ |
| Kanstrup, 2017 |  |  |  |
| Kanstrup, 2014 | ✓ |  |  |
| Kanstrup et al., 2010 |  |  |  |
| Kim et al., 2020 |  | ✓ | ✓ |
| Kwon and Ju, 2023 | ✓ |  |  |
| Lindskrog et al., 2019 |  |  | ✓ |
| Lobo et al., 2022 |  |  | ✓ |
| Lupton et al., 2017 | ✓ |  |  |
| Mariano et al., 2023 |  | ✓ |  |
| Martinez et al., 2016 |  |  |  |
| Merino-Barbancho et al., 2023 |  | ✓ |  |
| Montalva Colomer et al., 2014 | ✓ |  |  |
| Morand et al., 2023 | ✓ |  |  |
| Palmer et al., 2023 | ✓ |  |  |
| Rumeau et al., 2021 | ✓ |  |  |
| Samuelson et al., 2023 | ✓ |  |  |
| Santa et al., 2023 |  |  | ✓ |
| Snaphaan et al., 2022 |  | ✓ |  |
| Spinelli et al., 2019 | ✓ | ✓ |  |
| Swinkels et al., 2018 | ✓ | ✓ |  |
| Vaziri et al., 2016 |  |  | ✓ |
| Ward et al., 2015 |  | ✓ |  |

**Supplementary Table 7: LLs service outcomes**

| **Author (Year)** | **Research outcomes** | | | **Innovation Outcomes** | | | **Social and economic outcomes** | | | | |
| --- | --- | --- | --- | --- | --- | --- | --- | --- | --- | --- | --- |
|  | **Participatory Design** | **Real-World Testing** | **Interdisciplinary Collaboration** | **Improving Healthcare Service Quality & Patient Outcomes** | **Promoting Patient Engagement & Digital Health Literacy** | **Promoting E-health and Data-informed Health** | **Enhancing Healthcare Accessibility** | **Social Inclusion and Community Empowerment** | **Education and Training** | **Policy Development and Healthcare Governance** | **Business Development and Economic Growth** |
| Aharony et al. (2011) |  | ✓ |  |  | ✓ |  |  |  |  |  |  |
| Aguirre et al., 2016 |  |  |  |  | ✓ |  |  |  |  |  |  |
| Almeida et al. (2022) |  |  | ✓ |  | ✓ |  |  |  |  |  |  |
| Benvenuto et al., 2017 |  |  |  |  |  | ✓ |  | ✓ |  |  |  |
| Brauner et al. (2015) |  |  | ✓ |  |  | ✓ |  |  |  |  |  |
| Burrows et al., 2022 |  |  |  |  |  |  |  |  |  |  | ✓ |
| Byrne et al. (2023) |  | ✓ |  |  |  |  |  |  | ✓ |  |  |
| Busnatu et al., 2022 |  |  |  | ✓ |  |  |  |  |  | ✓ |  |
| Callari et al. (2019) | ✓ |  |  |  |  |  |  |  |  |  |  |
| Callari et al. (2020) | ✓ |  |  |  |  |  |  |  |  | ✓ |  |
| Cuomo et al. (2021) |  |  | ✓ |  |  | ✓ |  |  |  |  | ✓ |
| De Witte et al. (2021) | ✓ |  |  |  |  |  |  |  | ✓ |  |  |
| Dietrich et al., 2021 |  |  |  | ✓ |  |  |  |  |  |  |  |
| Fleet et al. (2020) |  |  | ✓ |  |  |  | ✓ |  | ✓ |  |  |
| Fotis et al. (2022) | ✓ |  |  |  |  |  |  |  | ✓ |  |  |
| Fotis et al. (2023) | ✓ |  |  |  |  |  |  |  |  | ✓ |  |
| Georges et al. (2016) |  | ✓ |  |  |  |  |  |  |  |  |  |
| Hamm et al. (2019) |  | ✓ |  |  | ✓ |  |  |  |  |  |  |
| Harvey and Jones, 2022 |  |  |  |  | ✓ | ✓ |  |  |  |  |  |
| Haukipuro et al. (2019) | ✓ |  |  |  | ✓ |  |  |  |  |  |  |
| Haukipuro & Vainamo (2019) |  | ✓ | ✓ |  |  |  |  |  |  |  |  |
| Haux et al. (2014) | ✓ |  |  |  |  |  |  | ✓ |  |  | ✓ |
| Harvey & Jones (2022) |  | ✓ |  |  |  |  |  |  |  |  |  |
| Hogervorst et al. (2023) | ✓ |  |  | ✓ | ✓ |  |  |  |  | ✓ |  |
| Kanstrup (2014) |  |  | ✓ |  |  |  |  |  |  |  |  |
| Kanstrup (2017) |  | ✓ |  |  |  |  |  |  |  |  |  |
| Kanstrup et al., 2010 |  |  |  | ✓ |  |  |  |  |  |  |  |
| Kim and Choi, 2023 |  |  |  |  | ✓ |  |  |  |  |  |  |
| Kim et al. (2020) |  |  | ✓ |  | ✓ |  | ✓ |  |  |  |  |
| Kim et al., 2021 |  |  |  |  | ✓ | ✓ |  |  |  |  |  |
| Kim et al., 2022 |  |  |  |  |  |  | ✓ | ✓ |  |  |  |
| Kwon & Ju (2023) |  | ✓ |  |  |  |  |  | ✓ |  |  |  |
| Leminen et al. (2017) |  |  | ✓ |  |  |  |  |  |  |  |  |
| Lepik & Krigul (2021) | ✓ | ✓ |  |  | ✓ |  |  |  |  |  |  |
| Lindskrog et al., 2019 |  |  |  | ✓ |  |  |  |  |  |  |  |
| Lobo et al. (2022) |  |  | ✓ |  |  | ✓ |  |  |  |  |  |
| Luengo-Polo et al. (2021) |  |  | ✓ |  |  |  |  |  |  |  |  |
| Lupton et al. (2017) | ✓ |  |  |  |  |  |  |  |  |  |  |
| Mariano et al. (2023) |  |  | ✓ |  |  | ✓ |  | ✓ |  |  |  |
| Martinez et al. (2016) |  |  | ✓ |  |  |  |  |  |  | ✓ |  |
| Merino-Barbancho et al. (2023) |  |  | ✓ |  |  | ✓ |  |  |  |  |  |
| Montalva Colomer et al. (2014) | ✓ |  | ✓ |  |  |  |  |  |  |  |  |
| Noublanche et al. (2019) | ✓ |  |  | ✓ |  | ✓ |  |  |  |  |  |
| Palmer et al. (2023) | ✓ |  |  |  |  |  |  |  |  | ✓ |  |
| Phanareth et al. (2017) |  | ✓ | ✓ |  | ✓ |  |  |  |  |  |  |
| Rahman et al. (2022) |  |  | ✓ | ✓ |  |  |  |  |  |  |  |
| Rumeau et al., 2021 |  |  |  |  |  |  |  | ✓ |  |  |  |
| Samuelson et al. (2023) | ✓ | ✓ |  | ✓ | ✓ |  |  |  | ✓ |  |  |
| Santa et al., 2023 |  |  |  |  | ✓ | ✓ | ✓ |  |  | ✓ |  |
| Schmitz et al. (2022) | ✓ |  |  |  | ✓ |  |  |  | ✓ |  |  |
| Snaphaan et al. (2022) |  |  | ✓ |  | ✓ |  |  | ✓ |  |  |  |
| Spinelli et al. (2019) | ✓ |  | ✓ | ✓ |  |  |  |  |  |  |  |
| Swinkels et al. (2018) | ✓ |  |  |  | ✓ |  | ✓ |  |  |  |  |
| Vaziri et al. (2016) |  | ✓ |  |  |  |  |  |  |  |  |  |
| Verbeek et al. (2020) | ✓ |  |  |  |  |  |  |  | ✓ |  |  |
| Ward et al. (2015) |  |  | ✓ |  |  |  |  |  |  |  |  |

**Supplementary Table 8: LLs outcomes measures**

| **Author(s)** | **Acceptability** | **Appropriateness** | **Effectiveness** | **Efficiency** | **End-user Satisfaction** | **Functionality** | **Safety** | **Equity** |
| --- | --- | --- | --- | --- | --- | --- | --- | --- |
| Aguirre et al., 2016 | ✓ | ✓ |  |  |  |  |  |  |
| Almeida et al., 2022 | ✓ |  |  |  |  |  |  |  |
| Benvenuto et al., 2017 |  |  | ✓ |  | ✓ | ✓ |  |  |
| Brauner et al., 2015 | ✓ |  |  |  |  |  |  |  |
| Busnatu et al., 2022 |  | ✓ | ✓ | ✓ |  |  |  |  |
| Callari et al., 2010 |  |  |  |  |  |  | ✓ |  |
| De Witte et al., 2021 |  |  |  |  |  |  |  | ✓ |
| Demiris et al., 2013 | ✓ |  |  |  |  |  |  |  |
| Hamm et al., 2019 |  |  | ✓ | ✓ | ✓ | ✓ |  |  |
| Haukipuro & Vainamo, 2019 | ✓ | ✓ |  |  |  |  |  |  |
| Hogervorst et al., 2023 |  |  | ✓ | ✓ | ✓ | ✓ |  |  |
| Kanstrup et al., 2010 | ✓ | ✓ |  |  |  |  |  |  |
| Kim et al., 2020 | ✓ | ✓ |  |  | ✓ | ✓ |  |  |
| Kim et al., 2021 |  |  | ✓ |  |  |  |  | ✓ |
| Lindskrog et al., 2019 |  |  | ✓ |  | ✓ | ✓ |  |  |
| Lobo et al., 2022 |  |  | ✓ | ✓ | ✓ |  |  |  |
| Mariano et al., 2023 | ✓ | ✓ |  |  |  |  |  |  |
| Merino-Barbancho et al., 2023 | ✓ | ✓ |  |  |  |  |  |  |
| Rahman et al., 2022 |  |  | ✓ | ✓ |  |  |  |  |
| Santa et al., 2023 |  |  | ✓ | ✓ | ✓ | ✓ |  | ✓ |
| Schmitz et al., 2022 | ✓ | ✓ |  |  |  |  |  |  |
| Seo et al., 2021 |  |  | ✓ | ✓ | ✓ | ✓ |  |  |
| Snaphaan et al., 2022 | ✓ | ✓ |  |  |  |  |  |  |
| Spinelli et al., 2019 |  | ✓ |  |  |  |  |  |  |
| Vaziri et al., 2016 |  |  | ✓ |  | ✓ |  |  |  |
| Ward et al., 2015 | ✓ | ✓ | ✓ |  |  |  |  |  |

**Supplementary Table 9: LLs challenges**

| **Authors** | **Accessibility & Usability** | **User Collaboration** | **Stakeholder Engagement** | **Financial Sustainability** | **Privacy & Ethics** | **Scaling & Policy Integration** |
| --- | --- | --- | --- | --- | --- | --- |
| Almeida et al., 2022 |  |  |  | ✓ |  |  |
| Benvenuto et al., 2017 |  |  |  |  |  | ✓ |
| Brauner et al., 2015 | ✓ |  |  |  |  |  |
| Busnatu et al., 2022 | ✓ |  |  |  |  |  |
| Byrne et al., 2023 |  |  |  |  | ✓ |  |
| Callari et al., 2019 |  |  |  |  | ✓ |  |
| Callari et al., 2020 |  |  |  |  | ✓ |  |
| Cuomo et al., 2021 |  |  |  | ✓ |  | ✓ |
| De Witte et al., 2021 |  | ✓ |  |  |  |  |
| Demiris et al., 2013 |  |  |  |  | ✓ |  |
| Dietrich et al., 2021 |  |  |  | ✓ |  |  |
| Harvey and Jones, 2022 |  |  | ✓ |  |  |  |
| Hogervorst et al., 2023 |  | ✓ |  |  |  |  |
| Kanstrup et al., 2010 |  |  |  |  | ✓ | ✓ |
| Kanstrup, 2014 |  | ✓ |  |  |  |  |
| Kim and Choi, 2023 |  |  | ✓ |  |  |  |
| Kim et al., 2022 |  |  | ✓ | ✓ |  |  |
| Lepik and Krigul, 2021 |  |  | ✓ | ✓ |  | ✓ |
| Lindskrog et al., 2019 |  |  |  | ✓ |  |  |
| Lobo et al., 2022 | ✓ | ✓ |  |  |  |  |
| Luengo-Polo et al., 2021 | ✓ | ✓ |  |  |  |  |
| Mariano et al., 2023 |  |  | ✓ | ✓ |  | ✓ |
| Morand et al., 2023 |  |  |  |  | ✓ |  |
| Noublanche et al., 2019 |  | ✓ |  |  |  | ✓ |
| Palmer et al., 2023 |  | ✓ |  |  |  |  |
| Rumeau et al., 2021 |  | ✓ |  |  |  |  |
| Samuelson et al., 2023 |  |  | ✓ |  |  |  |
| Santa et al., 2023 |  |  | ✓ |  |  |  |
| Seo et al., 2020 | ✓ |  | ✓ |  |  |  |
| Snaphaan et al., 2022 |  | ✓ |  | ✓ | ✓ |  |
| Vaziri et al., 2016 |  |  |  | ✓ |  |  |
| Verbeek et al., 2020 | ✓ |  | ✓ | ✓ |  | ✓ |
| Ward et al., 2015 |  |  |  |  |  |  |

**Supplementary Table 10: LLs Research priorities**

| **Author(s) & Year** | **Longitudinal Studies & Scalability** | **Scaling Methodologies** | **Economic Feasibility & Cost-effectiveness** | **AI-driven Healthcare Solutions** | **Inclusive Digital Health Strategies** | **Policy Development** |
| --- | --- | --- | --- | --- | --- | --- |
| Aharony et al., 2011 |  | ✓ |  |  |  |  |
| Almeida et al., 2022 | ✓ |  |  | ✓ |  |  |
| Benvenuto et al., 2017 |  |  | ✓ |  |  |  |
| Burrows et al., 2022 | ✓ |  |  |  |  |  |
| Busnatu et al., 2022 | ✓ |  |  |  |  |  |
| Byrne et al., 2023 |  |  |  |  |  | ✓ |
| Callari et al., 2019 |  |  |  |  |  | ✓ |
| Callari et al., 2020 |  |  |  |  |  | ✓ |
| Cuomo et al., 2021 |  | ✓ | ✓ |  |  |  |
| De Witte et al., 2021 |  |  |  |  |  | ✓ |
| Demiris et al., 2013 |  |  |  |  |  | ✓ |
| Dietrich et al., 2021 |  |  |  | ✓ |  |  |
| Fleet et al., 2020 | ✓ |  |  |  |  |  |
| Georges et al., 2016 | ✓ |  |  |  |  |  |
| Kanstrup, 2014 | ✓ |  |  |  |  |  |
| Kanstrup, 2017 |  |  |  | ✓ |  |  |
| Kanstrup et al., 2010 |  | ✓ |  |  |  |  |
| Kim and Choi, 2023 |  |  |  |  | ✓ |  |
| Kim et al., 2021 | ✓ |  |  |  | ✓ |  |
| Kim et al., 2022 | ✓ |  |  |  |  |  |
| Lindskrog et al., 2019 |  |  |  |  | ✓ |  |
| Luengo-Polo et al., 2021 |  |  |  | ✓ |  |  |
| Lupton et al., 2017 | ✓ |  |  | ✓ |  |  |
| Mariano et al., 2023 |  | ✓ |  |  |  |  |
| Merino-Barbancho et al., 2023 |  | ✓ |  |  |  |  |
| Montalva Colomer et al., 2014 |  |  |  |  |  | ✓ |
| Morand et al., 2023 |  |  |  |  |  | ✓ |
| Noublanche et al., 2019 |  | ✓ |  |  |  |  |
| Rahman et al., 2022 |  |  |  |  | ✓ |  |
| Seo et al., 2020 | ✓ |  |  |  | ✓ |  |
| Spinelli et al., 2019 |  |  | ✓ |  |  |  |
| Swinkels et al., 2018 |  |  |  | ✓ |  |  |
| Vaziri et al., 2016 |  |  | ✓ |  |  |  |
| Verbeek et al., 2020 |  | ✓ | ✓ |  |  |  |
| Ward et al., 2015 |  |  |  |  | ✓ |  |
